# Don't Think That Kids Aren't Noticing: Indirect Pathways to Children's Fear of COVID-19

**DOI:** 10.3389/fpsyg.2021.635952

**Published:** 2021-03-11

**Authors:** Ana Radanović, Isidora Micić, Svetlana Pavlović, Ksenija Krstić

**Affiliations:** ^1^Institute for Educational Research, Belgrade, Serbia; ^2^Laboratory for Developmental Psychology, Faculty of Philosophy, University of Belgrade, Belgrade, Serbia

**Keywords:** fear, COVID-19, children, parents, indirect pathways

## Abstract

The present study is couched within Rachman's three-pathway theory of fear acquisition (Rachman, 1977, 1991). Besides the direct contact with the objects of fear, this model also includes two indirect pathways to fear acquisition: negative information transmission and modeling. The study aims to explore the contribution of these three factors to the level of children's fear of COVID-19. The sample consisted of 376 children (59.6% girls), aged 7–19 (*M*_age_ = 12.77, SD_age_ = 3.57), and one of their parents (*M*_age_ = 42.88, SD_age_ = 6.00). The survey was conducted online during the COVID-19 national state of emergency in the Republic of Serbia. The children assessed their fear of COVID-19, general fearfulness, negative information transmission, and modeling by their parents, as well as the level of exposure to negative information outside their home. The parents assessed their own fear of COVID-19 and trait anxiety. Parents' anxiety, children's age, and children's general fearfulness were used as covariates. The results of our path analysis provide support for Rachman's notion of indirect pathways. The more the parents were afraid of COVID-19, the more they expressed this (either verbally or through their behavior), which in turn led to an increase in the children's fear of COVID-19. Furthermore, children's exposure to negative information related to COVID-19, provided by their teachers and peers or stemming from the media, directly contributed to the level of children's fear. The results of the study emphasize the importance of caregivers' behavior during global health crises and provide some clues as to what caregivers may do to protect their children's mental health in such circumstances.

## Introduction

The COVID-19 pandemic has raised many important questions related to children's coping mechanisms in stressful situations, as well as to their general psychological functioning during global health crises. Various studies conducted during the ongoing pandemic showed negative effects of the pandemic on children's mental health (Brown et al., 2020; Jiao et al., 2020; Orgilés et al., 2020; Pisano et al., 2020; Smirni et al., 2020). People's fears related to COVID-19 seem to be normative during the pandemic and have the adaptive function of inducing people to take care of themselves and others. However, the crisis is still ongoing, and it is not clear when it will end. Thus, normative fears of adults and children might develop into clinical fears that disrupt mental health not only during the crisis, but also afterwards.

Traditionally, a direct traumatic experience with the objects of fear was considered to be the dominant path to the development of clinical fears and phobias, as emphasized in the conditional model of fear development (Askew and Field, 2008). This model assumes that people associate a neutral stimulus with traumatic events, which then leads to a fear reaction. As a result, a previously neutral stimulus starts to elicit a fear reaction by itself. Although this pathway to fear development is empirically well-supported (Field and Davey, 2001), already 30 years ago Rachman (1977) noticed that some other factors, not related to traumatic experiences, also contribute to the development of children's fears. Rachman (1977, 1991) formulated his three-pathway model, which, besides direct conditioning, also includes indirect learning processes. These indirect pathways to children's fears include observing fearful reactions of people around the child (vicarious learning or modeling, see Askew and Field, 2008, for more details) and negative information or instructions related to the objects of fear (negative or threat information transmission). These indirect pathways can also trigger the acquisition of fear [see Muris and Field (2010), for more details] or anxiety (Percy et al., 2016). Rachman's model has gained wide empirical support [see King et al. (1998), for details] and has been incorporated into new models of fear acquisition [e.g., Field and Davey (2001) and Muris and Merckelbach (2001)]. However, one of the main methodological issues raised about the empirical validation of Rachman's model is the validity of retrospective accounts. In order to overcome this issue and be able to study the effects of negative information transmission in more controlled conditions, researchers have developed a prospective paradigm (Field et al., 2001) and conducted experimental studies, which also supported Rachman's theory about the role of the indirect pathways in the development of fears.

Furthermore, Rachman's model was used as the theoretical framework in the study of children's fear of Swine Flu (Remmerswaal and Muris, 2011) in a naturalistic context and with real-time assessment of fear during the peak of the 2009 Swine Flu pandemic. Like the 2009 Swine Flu pandemic, the COVID-19 pandemic is a public health crisis during which heightened levels of fear are experienced. The COVID-19 pandemic is therefore another naturalistic context for further exploration of children's fears and the influence of the behavior of people around them on developing or increasing their fears during global health crises.

Various studies have shown that children's fears can start developing within the family context, and that, if not recognized and treated, growing fears can lead to psychopathology later in life. Communication with children is important for fear management [e.g., Rapee et al. (2009)]. Consequently, the role of parents' behavior is the most frequently explored factor in the development of children's specific fears. Parents' abilities to efficiently manage their fears and cope with stress are especially crucial during crisis periods such as the COVID-19 pandemic [e.g., Duan et al. (2020)], not only when it comes to their own well-being, but also when it comes to the well-being of their children. The results of a recent study by Spinelli et al. (2020) have shown that parents who were dealing with more difficulties related to the COVID-19 lockdown conditions reported a higher level of stress, which in turn increased their children's stress levels. Parents' medical fears such as dental fear [e.g., Tahmourespour et al. (2014)] or fear of a specific disease [e.g., Remmerswaal and Muris (2011)] have been shown to correlate significantly with these fears among their children.

The current study is based on the previously mentioned correlation study about children's fear of Swine Flu. In this study, Remmerswaal and Muris (2011) investigated the contribution of negative (threat) information (provided by parents) to children's fear of Swine Flu during the peak of the 2009 Swine Flu pandemic in the Netherlands. The results of their study showed that parents' threat information partially mediated the correlation between the parents' and children's fear of Swine Flu. This is one of many studies that supported Rachman's idea that negative information and threat narratives may be a risk factor for children's fear. Furthermore, the experimental study conducted by Muris et al. (2010) also showed that parents could induce children's fear beliefs by providing threatening narratives related to the object of fear.

In addition to the indirect pathway of verbal threat information transmission, on which the study about Swine Flu focused, the current study also includes another indirect pathway related to observing others' fearful reactions, namely modeling. Askew and Field (2008) showed that fear can be transmitted even in the absence of direct contact or verbal threat information. They conducted an experimental study that demonstrated that children's fear increased for novel animals which they saw paired with scared faces. It also took longer for the children to approach a box with the animals they had previously seen paired with scared faces. Furthermore, Gerull and Rapee (2002) showed that children expressed greater fear and avoidance of stimuli, which were followed by their mothers' adverse reaction.

Our study includes children's real-time assessments of their parents' fearful reactions during the COVID-19 national state of emergency. This indirect pathway may be particularly important in a pandemic. Even if parents try to hide their fear in the verbal communication with their children in order to protect them, non-verbal communication is more difficult to control because it is not always conscious or planned.

The third indirect pathway included in the current study was threat information to which the children were exposed outside the communication with family members, for instance, in interactions with their peers and teachers, on the internet, or in the news. This pathway is particularly important for our study because of the specific circumstances of the COVID-19 pandemic, including online schooling and frequent news reports about COVID-19 on television and on the internet. Due to all these factors, during the COVID-19 national state of emergency, children may have been exposed to a vast amount of negative information concerning the COVID-19 pandemic, and it is therefore vital to examine the contribution of this pathway to the levels of children's fear of COVID-19.

Furthermore, there are important factors that may interfere with the connection between parents' and children's fear. Anxiety, by its very nature, implies increased fear of many different things and parents with higher levels of anxiety may be more afraid of COVID-19. In addition, parents' anxiety levels may affect their children's fear of COVID-19. Muris et al. (2010) established the important role of parents' trait anxiety level in children's fear. After receiving ambiguous information related to the object of fear, parents who scored higher on anxiety told their children more negative stories related to the object of fear, which in turn led to higher fear levels in their children. An insightful study by Remmerswaal et al. (2016) showed that a brief training by parents can influence their children's information search bias. Children who received negative training by the parent (meaning that the parents were instructed to encourage their children to search for negative information) exhibited an increase in negative information search, as well as in fear. Parents who are more anxious and more afraid of COVID-19 are more focused on searching for negative information, which their children may notice in everyday settings. In addition to the correlation with modeling and negative information transmission, parents' anxiety and fear level may also be associated with the children's exposure to negative information related to COVID-19 outside the communication with family members. Children's age is also an important factor for fear regulation. Previous research showed that children's levels of fears decreased with age [e.g., Gullone and King (1997)]. Furthermore, a preliminary study conducted in the Shaanxi Province of China during the COVID-19 pandemic showed that younger children aged 3–6 were more likely than older children to manifest the fear that someone in the family might have the infection (Jiao et al., 2020). Finally, it is plausible to expect that children's general fearfulness is associated with their specific fear of COVID-19, which can be considered one of the medical fears incorporated in children's general fearfulness. Since all the mentioned factors are associated with fear acquisition in children, they are included in this study as covariates of the primary variables of the study.

Our research was conducted during the national state of emergency in the Republic of Serbia that was declared on March 15, 2020. Universities, schools, preschools, and nurseries were closed, as well as the state borders. Frequent curfews during evening hours and weekends were introduced as one of many measures in order to slow down the spreading of COVID−19. Information on the numbers of the infected and the deceased, as well as new prevention and prohibition measures were often broadcast on all TV channels. Since the mortality rate is the highest among the oldest, citizens were urged not to visit their elderly parents and not to take their children to visit their grandparents. Those older than 65 were instructed not to leave their residences during the national state of emergency. School classes were broadcasted on national TV channels. Considering all measures taken by the authorities, the children spent their time mainly in the family home with more limited live interactions with their peers and other important figures.

Based on the same theoretical framework as the previously described studies of fear acquisition pathways, our study aims to explore children's fear of COVID-19 during the national state of emergency in Serbia, in the light of Rachman's three-pathway model, controlling for parents' anxiety, children's age, and children's general fearfulness. The study's main hypothesis is that children's fear of COVID-19 may be connected with their parents' fear of COVID-19 through parents' fearful reactions and verbal transmission of negative information related to COVID-19. Our main hypothesis is that information transmission and modeling will partially or fully mediate the relationship between parents' and children's fear of COVID-19, while conditional learning and negative information to which children are exposed outside the family will affect children's fear of COVID-19 directly. Since the COVID-19 crisis is particular in many respects, our study needs to remain more exploratory than explanatory in nature.

## Method

### Participants

The initial database consisted of 1,412 parent–child dyads. As a first step, all incomplete questionnaires and questionnaires completed only by parents or only by children were removed from the database. After this step, a total of 378 dyads remained. As an additional selection criterion, the questionnaires, for both the children and the parents, ended with a question related to answering the questions honestly (“*It is very important to us to know if you answered all the questions honestly. Please select the answer below which describes your answers most accurately*”). All the children reported that they answered the questions *mostly* or *completely honestly*. However, two parents reported that they answered the questions *completely dishonestly*. At the second step, the data obtained from these participants were excluded from the study (together with the data obtained from their children). The final sample therefore included 376 children (59.6% girls), aged 7–19 (*M* = 12.77 years, SD = 3.57), and one of their parents (*n* = 376), aged 27–67 (*M* = 42.88 years, SD = 6.00). During the lockdown, 35.1% of the parents worked from home, while the remainder either worked outside home (17.6%), sometimes at home and sometimes outside home (13.0%), or did not work at all (34.3%).

### Procedures

Due to the COVID-19 pandemic, a national state of emergency was declared in Serbia, including lockdown, school closures, and frequent curfews. Therefore, the only possible way to collect data was through an online survey. We launched a survey via SoSci Survey (Leiner, 2020). The survey was available during the national state of emergency (from April 16 to May 6, 2020) and took ~20 and 15 min for children and parents to complete, respectively. The invitation for participation in the study was distributed via social networks. Information on the study was presented to the parents, requesting their consent for their and their child's voluntary and anonymous participation in the study. If they had more than one child aged 7–19, we asked them to participate in the study with the younger child. If the parents had agreed to participate, the children were asked to complete the questionnaires first. It should be noted that we strongly advised the parents not to interfere with their children's responses, emphasizing the importance of obtaining the children's independent responses for the study. However, we did ask the parents to help younger children complete the questionnaire if they had some technical issues or if they struggled to understand the questions. The final part of the questionnaire contained posters with information related to interesting home activities, as well as online educational materials for children, along with useful information for parents about children's reactions to crisis situations, which may occur during the lockdown and different help methods which parents can use to teach their children how to cope with stress.

### Measures

#### Children

*The Fear of COVID-19 Questionnaire for Children* (FC19Q-C) was constructed for the current study to measure children's fear related to COVID-19 (Supplementary Appendix A). The measure consisted of 14 items in total, out of which three items were modified from the Fear of Swine Flu Questionnaire (henceforth FSFQ), which was constructed to measure children's fear of Swine Flu during the 2009 Swine Flu Pandemic (Remmerswaal and Muris, 2011). Cronbach's alpha for FSFQ reported by Remmerswaal and Muris is 0.81. The items from the FSFQ used in the present study are the following: “*Would you be scared if you had the coronavirus?*,” “*Are you more afraid to become ill since the coronavirus's outbreak?*” and “*Would you be scared if someone you know had the coronavirus*?” In accordance with the suggestion to use a simpler answering format for children (Brasic Royeen, 1985; Mellor and Moore, 2014), younger children (7–11 years old) were asked to answer the questions using a three-point Likert scale ranging from 1 (*false*) to 3 (*true*), while adolescents (12–19 years old) had to answer the questions using a five-point Likert scale ranging from 1 (*strongly disagree*) to 5 (*strongly agree*). The answers on the three-point scales were weighed and transposed on the five-point scale, after which the mean FC19Q-C score was computed. A higher score indicates a higher level of COVID-19 fear. The internal consistency of the two subscales is satisfactory (Table 1).

**Table 1:** Descriptive statistics, reliability coefficients, and intercorrelations for study variables.

**Variable**	**M**	**SD**	**α**	**1**	**2**	**3**	**4**	**5**	**6**	**7**	**8**	**9**
Children												
1. Age	12.77	3.57	–	–								
2. Fear of COVID-19	2.82	0.83	0.85	−0.14^**^	–							
3. Fearfulness	2.46	0.68	0.83	−0.21^**^	0.41^**^	–						
4. Modeling	2.33	0.77	0.71	0.13^*^	0.42^**^	0.29^**^	–					
5. Family Transmission	3.41	1.18	0.63	0.06	0.38^**^	0.21^**^	0.45^**^	–				
6. Non-Family Transmission	2.40	0.83	0.58	0.38^**^	0.36^**^	0.14^**^	0.35^**^	0.19^**^	–			
Parents												
7. Fear of COVID-19	2.73	0.74	0.86	0.03	0.49^**^	0.32^**^	0.55^**^	0.36^**^	0.20^**^	–		
8. Cognitive Anxiety	1.70	0.63	0.87	0.06	0.17^**^	0.27^**^	0.37^**^	0.12^*^	0.21^**^	0.44^**^	–	
9. Somatic Anxiety	1.40	0.53	0.89	0.08	0.19^**^	0.19^**^	0.36^**^	0.16^*^	0.19^**^	0.37^**^	0.69^**^	–

* p < 0.05;

** *p < 0.01*.

We tested Rachman's model using several questions for the assessment of direct experience with the object of fear. Firstly, we asked the participants if they had been infected (“*Have you been infected with the coronavirus?*”). Since the children could have had direct experience with COVID-19 in case of illness of their parents or siblings as well, we also asked them if someone in their family had been infected with COVID-19. Furthermore, we used two short measures for the assessment of the other two pathways of fear acquisition: negative information transmission and modeling (Supplementary Appendix B). We constructed the Non-Family Information Transmission Scale (NFITS) to assess the level of negative (threat) information to which the children have been exposed outside the family, from their teachers, peers, on TV or on the Internet. In addition, we created the Family Information Transmission Scale (FITS), which included three items related to parents' threat information. This scale was constructed following the model of the Sources of Information about the Swine Flu Scale (SISFS, Remmerswaal and Muris, 2011). Cronbach's alpha for the parents' threat information scale reported by Remmerswaal and Muris was 0.79. Finally, we constructed the Modeling Scale (MODS), which included nine items related to different fearful reactions and behaviors of the parents that their children may have observed during the COVID-19 pandemic. For all described instruments, the younger children used a three-point Likert scale ranging from 1 (*never/rarely*) to 3 (*every day*) to answer, while the older children used a five-point Likert scale ranging from 1 (*never*) to 5 (*every day*). The answers on the three-point scales were weighed and transposed on the five-point scale. Higher scores indicate higher levels of negative information transmission or higher level of modeling. The internal consistency of these scales is satisfactory (Table 1).

We modified the shortened version of the *Fear Survey Schedule for Children* (FSSC-R; Ollendick, 1983) to measure children's fear of specific situations and stimuli. This questionnaire is a widely used self-report measure via which specific fears and general fearfulness in youths can be measured. Our modified questionnaire included four items related to fears of small animals (e.g., spiders), four items related to the fear of danger and death (e.g., being hit by a car or a truck), three items related to the fear of failure and criticism (e.g., being teased), four items related to the fear of the unknown (e.g., the dark), and four items related to medical fears (e.g., getting a shot from the doctor). In addition to these 19 items, which were presented to both the younger and the older children, five more items were added to the questionnaire presented to the older children. This was done due to the difference in specific fears between younger and older children [e.g., Bauer (1976)]. Following previous studies about adolescents' fears [e.g., Ollendick and King (1994), Lane and Gullone (1999), and Michalčáková et al. (2009)], these additional items were created to address the fear of being abandoned (e.g., being left behind by my friends) and the fear related to identity (e.g., something is wrong with me).

We asked the children to indicate their fear level on a three-point Likert scale ranging from 1 (*not afraid*) to 3 (*afraid*) for the younger children and on a five-point Likert scale ranging from 1 (*not afraid at all*) to 5 (*very afraid*) for the older children. As for the FC19Q-C, the answers on three-point scales were weighed and transposed on the five-point scale. The score of the children's fears was obtained as a mean value of the responses across all 19 items for the younger children and all the 24 items for the older children. A higher score indicates a higher level of general fearfulness. The internal consistency of this questionnaire is satisfactory (Table 1).

#### Parents

*The Fear of COVID-19 Questionnaire for Parents* (FC19Q-P) is a modified version of the FC19Q-C used to assess parents' fears related to COVID-19. This questionnaire has the same 14 items as the FC19Q-C, adapted for adults, which need to be answered on a five-point Likert scale ranging from 1 (*strongly disagree*) to 5 (*strongly agree*). The internal consistency of the FC19Q-P is satisfactory (Table 1).

To measure the parents' trait anxiety, we used *The State-Trait Inventory for Cognitive and Somatic Anxiety* (*STICSA;* Ree et al., 2000). We used only the trait version of this inventory (measuring to what extent, in general, the statement is true for the participants) to measure 10 cognitive symptoms of anxiety (e.g., “*I cannot get some thought out of my mind*”) and 11 somatic ones (e.g., “*My muscles are tense*”). To answer, the participants used a four-point Likert scale ranging from 1 (*not at all*) to 4 (*very much so*). Cronbach's alphas for the inventory pertaining to cognitive and somatic symptoms of anxiety are satisfactory (Table 1).

## Results

### Analytic Plan

A path analysis was conducted to examine the associations between the children's fear of COVID-19, the parents' fear of COVID-19, and the direct and indirect pathways of fear acquisition. SPSS (version 26) was used for descriptive statistics and correlation analysis, whereas Amos (version 22) was used for conducting path analysis (Arbuckle, 2013). As a direct conditioning measure, we asked the children if they or someone in their family had been infected with COVID-19. Only three participants (0.8%) reported that they had been infected, and six of them (1.6%) reported that someone in their family had been infected with COVID-19. Due to the small number of children who directly experienced COVID-19, conditional learning as a direct pathway to fear was excluded from the analysis.

Considering the exploratory nature of our study and the study's primary goal to explore the mechanisms of children's acquisition of fear related to COVID-19, we started from the full, saturated path model. The model included the following primary study variables: Parent's fear of COVID-19, Children's fear of COVID-19, Non-family information transmission, Family information transmission, and Modeling. The latter three were used as measures of pathways included in children's fear acquisition.

The model fit was assessed by examining the comparative fit index (CFI; Marsh and Hau, 2007) and the Tucker–Lewis index (TLI; Bentler, 1990). With values >0.95, both indicate a good model fit. Further, the root mean square error of approximation (RMSEA; Hu and Bentler, 1999) with a value <0.05 and the *p*-value 0.000 indicate a good model fit.

A bias-corrected bootstrapping procedure (10,000 draws) was used for estimating the standard error and confidence interval for the total, direct, and indirect effect of Parents' fear of COVID-19 on Children's fear of COVID-19 through Non-family information transmission, Family information transmission, and Modeling. This approach was chosen since it generates the most accurate confidence intervals for the estimates of the effects, reducing Type I error rates and increasing power over other similar tests (MacKinnon et al., 2002). The model was trimmed according to the path analysis results. Non-significant paths were excluded one by one in a backward fashion.

### Selections of Covariates

Since previous studies had shown that parents' trait anxiety level could intensify negative information transmission (Muris et al., 2010; Remmerswaal et al., 2016), Parents' somatic anxiety and Parents' cognitive anxiety were selected as covariates of both Parents' fear of COVID-19 and Children's fear of COVID-19. Similarly, since children's fears have a normative, developmental path and change with age (Gullone and King, 1997), the measures of Children's general fearfulness and Children's age were defined as covariates of Parents' anxiety, Parents' fear of COVID-19, Children's fear of COVID-19, as well as of the three pathways of transmission. Since Child gender showed no correlation with Children's fear or any of the fear acquisition pathways, the same model was analyzed with the whole sample. All the above-mentioned covariates (Parents' somatic anxiety, Parents' cognitive anxiety, Children's general fearfulness, and Children's age) were introduced in the initial, saturated model, assuming their direct effect on the primary study variables. In the final model, only those with significant associations with any of the relevant variables were included.

### Model Results

**Figure 1:**
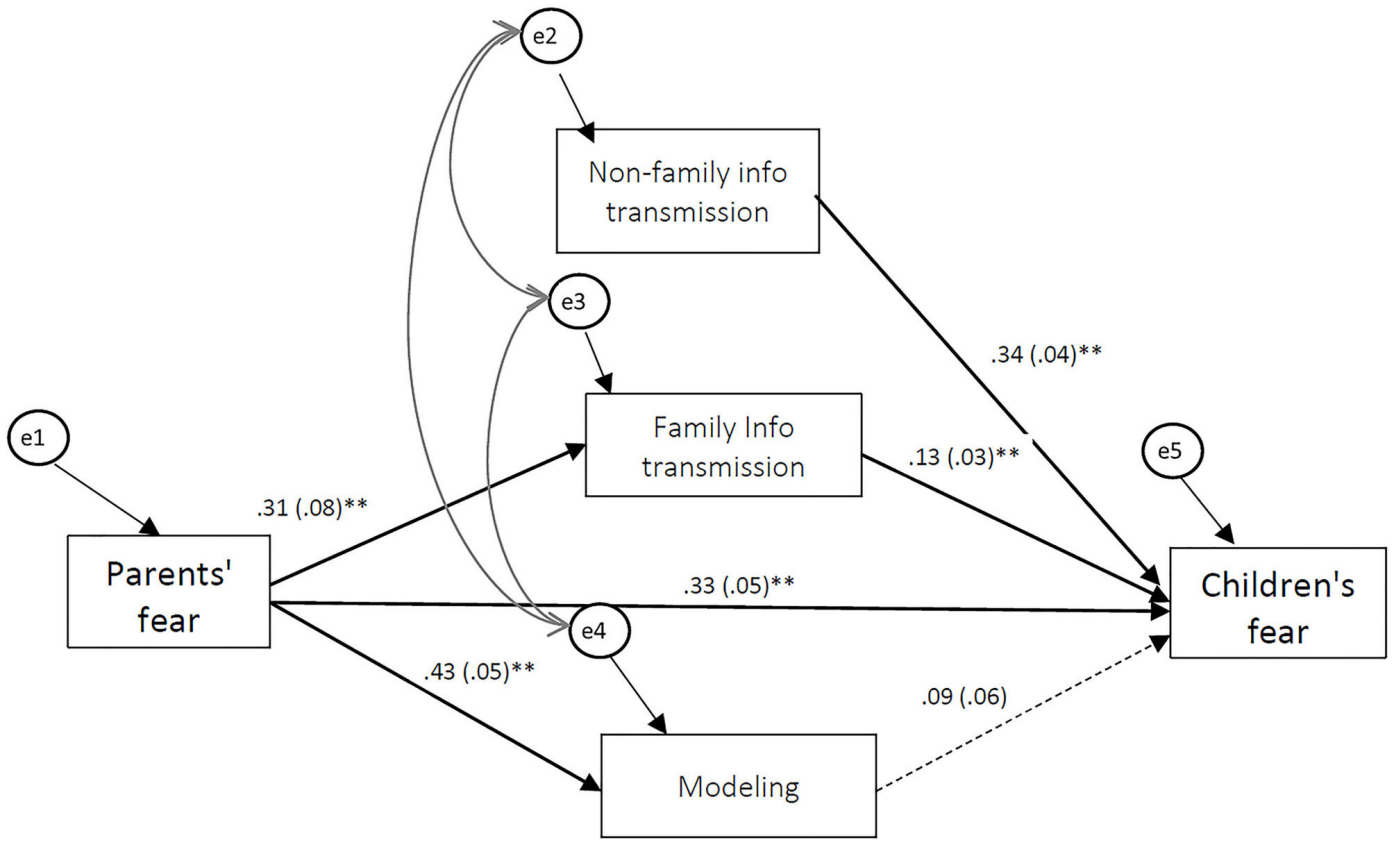
Path Model Predicting Children's Fear of COVID-19. Statistics are standardized regression coefficients. Children's age, general fearfulness, and parental anxiety were used as covariates but are not depicted. The dotted path is marginally significant, significant paths are bolded. **p < 0.01.

Descriptive statistics and correlations for the study variables are presented in Table 1. As expected, Children's fear of COVID-19 is correlated with Children's general fearfulness and negatively correlated with Children's age. Children's fear of COVID-19 is also correlated with Parent's fear of COVID-19, Parent's anxiety, and all the examined pathways of fear acquisition. On the other hand, Parents' fear of COVID-19 is correlated with Parent's cognitive anxiety, Parents' somatic anxiety and all three fear acquisition pathways. Non-family information transmission, Family information transmission, and Modeling are all correlated with Children's age and Children's general fearfulness, as well as with Parents' trait anxiety. The model analysis showed that the hypothesized model (Figure 1) fits the empirical data [χ^2^ (5, *n* = 376) = 7.316, *p* > 0.05, χ^2^/df = 1.463, CFI = 0.997, TLI = 0.982, RMSEA = 0.035, (CI = 0.000, 0.086)]. The standardized regression coefficients are shown in Table 2. As Table 2 shows, a Standardized Total Effect of Parents' fear of COVID-19 on Children's fear of COVID-19 is 0.410 (CI = 0.320, 0.498), while the direct effect of Parents' fear is 0.334 (CI = 0.233, 0.433). The total indirect effect of Parent's fear on Children's fear through Negative information transmission and Modeling is 0.076 (CI = 0.031, 0.129).

**Table 2:** Standardized regression coefficient for regression model.

**Relations**			**Standardized regression weights**	***p***
Parental cognitive anxiety	—>	Parents' fear of COVID-19	0.379	^***^
Children's general fears	—>	Parents' fear of COVID-19	0.223	^***^
Parents' fear of COVID-19	—>	Family information transmission	0.313	^***^
Parents' fear of COVID-19	—>	Modeling transmission	0.427	^***^
Children's general fears	—>	Non-family information transmission	0.190	^***^
Children's general fears	—>	Family information transmission	0.113	0.026
Children's general fears	—>	Modeling transmission	0.150	0.001
Children's age	—>	Non-family information transmission	0.419	^***^
Children's age	—>	Modeling transmission	0.154	^***^
Parental cognitive anxiety	—>	Non-family information transmission	0.145	0.002
Parental cognitive anxiety	—>	Modeling transmission	0.150	^***^
Parents' fear of COVID-19	—>	Children's fear of COVID-19	0.334	^***^
Non-family information transmission	—>	Children's fear of COVID-19	0.340	^***^
Family information transmission	—>	Children's fear of COVID-19	0.126	0.004
Modeling transmission	—>	Children's fear of COVID-19	0.086	0.091
Parental cognitive anxiety	—>	Children's fear of COVID-19	−0.135	0.002
Children's specific fears	—>	Children's fear of COVID-19	0.193	^***^
Children's age	—>	Children's fear of COVID-19	−0.242	^***^

*** *p < 0.001*.

Parents' fear of COVID-19 is directly related to Children's fear of COVID-19 (β = 0.334, *p* < 0.001). In addition to this direct connection, Parents' fear also indirectly influences Children's fear through two pathways of transmission. On the one hand, Parents' fear of COVID-19 is associated with Family information transmission (FITS) (β = 0.313, *p* < 0.001) and with Modeling (MODS) (β = 0.427, *p* < 0.001). On the other hand, Family information transmission and Modeling are associated with children's fear of COVID-19 (β = 0.126, *p* < 0.004; and β = 0.086, *p* < 0.091, respectively). However, the third indirect pathway, Modeling, is approaching statistical significance, so this finding should be verified in future research. This implies that the fear transmission mechanisms mediate the relation between Parents' and Children's fear of COVID-19. Parents who were on the average more afraid of COVID-19 passed more of their fear onto their children through verbal (and behavioral) mechanisms, which in turn resulted in their children being, on average, more afraid of COVID-19.

In addition to these family influences, Children's fear of COVID-19 is also associated with Non-family information transmission (NFITS) (β = 0.340, *p* < 0.001), meaning that those children who were more exposed to external threatening information showed a higher level of fear regarding COVID-19.

The examination of covariate effects is also significant in the interpretation of this model. Parents' somatic anxiety and Parents' cognitive anxiety were tested as covariates of both Parents' and Children's fear of COVID-19. Parents' somatic anxiety has no significant effects. On the other hand, Parents' cognitive anxiety not only affects Parents' fear of COVID-19 (β = 0.379, *p* < 0.001) but also has a small, direct effect on Children's fear of COVID-19. Interestingly, its effect on Children's fear is negative (β = −0.135, *p* < 0.002), indicating that the more anxious the parents were, the less afraid their children were. This finding should be verified in further studies. In addition, Parents' cognitive anxiety is associated with two pathways of fear transmission, Non-family information transmission (β = 0.145, *p* < 0.002) and Modeling (β = 0.150, *p* < 0.001).

As expected, Children's age is associated negatively with Children's fear of COVID-19 (β = −0.242, *p* < 0.001), indicating that as the age of the child increased, the fear of COVID-19 tended to decrease. Further, Children's age influences Non-family information transmission (β = 0.419, *p* < 0.001), as well as Modeling transmission (β = 0.154, *p* < 0.001), indicating that as children grow older, they are more exposed to information outside home and are therefore more susceptible to modeling influence.

Children's general fearfulness has significant, although small, effects on both Children's (β = 0.193, *p* < 0.001) and Parents' fear of COVID-19 (β = 0.223, *p* < 0.001), as well as on all the pathways of fear acquisition (Non-family information transmission, β = 0.190, *p* < 0.001; Family information transmission, β = 0.113, *p* < 0.026, and Modeling, β = 0.150, *p* < 0.001). These findings suggest that children who are generally more fearful are more afraid of COVID-19, and also have parents who are more afraid of COVID-19. In addition, their fear is affected by both pathways of fear transmission.

## Discussion

This study aimed to explore children's fear of COVID-19 in the light of Rachman's model of fear acquisition. We hypothesized that conditional learning would directly affect children's fear and that children's fear would be significantly influenced by parental fear, transmitted indirectly through two pathways, namely, negative information transmission, and modeling. Since the number of children who reported direct experience with COVID-19 as an object of fear was low, we had to exclude direct conditioning from further analysis. Thus, our study is based on the part of Rachman's model related to the indirect pathways of fear acquisition.

We hypothesized that parental trait anxiety, children's age, and children's general fearfulness might be important to control for because of their association with the variables regarding parents' and children's fear of COVID-19. Indeed, the relations between Children's fear of COVID-19, Children's age, and Children's general fearfulness are significant. As in previous studies [e.g., Jiao et al. (2020)], our results have shown that children's fear of COVID-19 decreases with age. Higher levels of children's fear of COVID-19 were associated with higher levels of their general fearfulness, indicating that children's fear of COVID-19 can be considered one of the medical fears among children's specific fears (Ollendick, 1983).

As expected, higher levels of parents' fear of COVID-19 were associated with higher levels of their fearful reactions and negative information transmission. These results are in line with previous studies [e.g., Remmerswaal and Muris (2011)] about other parents' specific fears, which lead to the transmission of negative (threat) information verbally by instruction or conversation, as well as by fearful behaviors (modeling). The consistency of these findings indicates that this effect is relatively independent of the object of fear and the context.

Interestingly, in a bivariate analysis, higher levels of parents' fear and cognitive and somatic anxiety were positively correlated with the children's exposure to negative information related to COVID-19 outside home. Despite this observed trend, parents' fear did not predict negative information transmission outside the communication with family members. However, parents' cognitive anxiety did show such an effect. Remmerswaal et al. (2016) showed that when mothers were instructed to direct their children toward searching for negative information (which we assume parents who scored higher on fear and anxiety did), their children displayed an increase in searching for negative information and in fear. In line with these findings, we assume that the children of the parents who scored higher on anxiety and fear of COVID-19 were more often searching for negative information about COVID-19 outside their home. However, it is also possible that, regardless of the children's willingness to be exposed to negative information about COVID-19, they were exposed to it, not in communication with their parents but still inside their home. For instance, the parents with increased fear and anxiety may have been following the news about COVID-19 more frequently. This is indeed quite likely, given the fact that during the COVID-19 national state of emergency in Serbia, daily media conferences with medical experts and politicians were held and broadcast live.

We defined the path model in order to test our central hypothesis and explore whether, as predicted by Rachman's theory, the parents' fear of COVID-19 led to an increase in the children's fear of COVID-19 through the indirect pathways of negative information transmission and modeling. The strongest predictor of children's fear of COVID-19 is parental fear of COVID-19. Thus, our model only partially explains the mechanisms of children's fear acquisition. Based on our findings, it cannot be explained in which way, i.e., via what mechanism, the parental fear directly impacts the children's fear of COVID-19. One possibility is that this study did not cover some of the indirect transmission mechanisms. On the other hand, there may be some direct paths, related to more basic mechanisms, such as the mechanism of emotional contagion, by which the parents' fear directly affects the children's fear. These hypotheses should be investigated in future research.

The significant indirect effect of parental fear on children's fear through negative information transmission and modeling indicates that parents who are more afraid tend to express their fear verbally or through their behavior, leading to an increase in children's fear. Hence, our starting hypothesis about the mechanisms of indirect transmission of parent's fear to children has been partially confirmed. Accordingly, these results support Rachman's theory (Rachman, 1977, 1991) about indirect fear acquisition pathways. The study by Remmerswaal and Muris (2011) about the fear of Swine Flu also showed that the parents' negative information transmission only partially mediated the correlation between the parents' and the children's fear of Swine Flu.

It should be noted that only a very small amount of variance of children's fear could be accounted for by the indirect pathways. This suggests that, in addition to the significant direct contribution of parental fears, there are other pathways, i.e., learning experiences associated with children's fear acquisition. Some authors discuss more complex models and multifaceted etiologies of children's fears and anxiety, including stressful life events, parental rearing behaviors, parental emotion regulation, existing beliefs, and expectations about the possible consequences [e.g., Muris and Merckelbach (2001)]. Subsequent research should examine the role and importance of these characteristics attributed to the family, the parents, and the child. Furthermore, when a crisis has been ongoing for some time, this means that children have been exposed to many sources of information (and disinformation) available at home and outside home. As a consequence, the pathway of negative information transmission outside the family may gain in importance, especially for older children, to whom this information is more accessible. Despite a small indirect effect, our findings indicate that both parental verbal messages and patterns of behavior (modeling) make a significant contribution to the onset of children's fears. In interpreting the effect size of these associations, it should be borne in mind that the data were obtained from two sources (from parents and children). It seems plausible that if all variables in the model had been measured based on parents' self-report, their associations would have been higher.

In interpreting the results, both the moment and the context in which the study was conducted should be considered. Although rigorous measures were introduced in order to prevent the spread of the pandemic, the children's and the parents' fear of COVID-19 was moderate (2.8 and 2.7, respectively, on a scale of 1–5). At the time, it seemed that the epidemic was under control, that the spread of the virus could be contained, and there was a small number of the infected and the deceased. All these factors could have impacted not only the fear of the parents but also their behavior toward and verbal messages to the children. Fitzpatrick et al. (2020) reported similar results about the level of fear of COVID-19. They conducted an online survey in March 2020 on a nationally representative sample of adults in the United States. Their results showed that on a scale from 1 (not at all fearful) to 10 (very fearful) the average scores ranged from 6.8 to 7.2, depending on the region. However, it would be interesting to study the effects of the examined constructs in a more uncertain situation, less under the individual's control, as well as within medical or government systems. Further studies should consider broader contextual factors, which can affect both the parents and the family system and have a direct impact on children's fears.

### Implications

For most children, different fears, the medical ones included, are a common, normative part of their childhood and adolescence. However, some children have trouble dealing with intense fears, which makes their daily functioning more difficult (Muris et al., 2000). In times of crisis, fears tend to intensify, especially those related to the causes of the crisis. Therefore, during an ongoing COVID-19 pandemic, attention should be paid to children's fears related to COVID-19 and the potential risk factors that may intensify and prolong those fears. This study showed the importance of the parents' role during a global health crisis, and it serves as a warning that children are learning even when parents are unaware of it. The results emphasize the importance of the way we communicate with our children in a period of crisis. Since threatening narratives increase children's fears, approaching their concerns differently could be more beneficial, as it may help the children understand the pandemic and positively affect their well-being. To influence children's perspective positively and reduce the intensity of their worries, it may be useful to focus on the positive aspects of a pandemic situation, e.g., more time to play with parents or siblings, various interesting activities that the child can engage in at home, or exploring new ways of schooling. It seems important to send the children a message that they are safe and that the family is taking care of them, as well as to teach them to follow health experts' guidelines, thereby participating in this “battle against the virus.” In addition to verbal communication, the results of this study show the importance of behaviors that are not necessarily followed by a verbal message. Parents' non-verbal reactions to the news about the daily numbers of people infected or non-verbal signs of panic when someone is leaving the house can signal danger to the child on a much larger scale than desired. Some authors emphasize the importance of the dynamics of the family system and mutual communication among its members during an ongoing pandemic [e.g., Prime et al. (2020)]. Children's adaptation to the pandemic conditions, as well as their coping with the psychological consequences (which may even come after the pandemic), depend on many factors, but the family environment is among the most important ones. It should be emphasized that the indirect pathways related to negative information transmission by peers, teachers, the news, or other sources may also increase children's fear. Parents need to be informed about the information their children are exposed to from different sources and discuss it with their children in order to diminish their concern or prevent them from misunderstanding such information. One of the most important suggestions for the parents is to take care of themselves in the first place. Given all the health-related and economic concerns that parents face daily during the ongoing pandemic, it cannot be expected that all parents will always respond to the new challenges in the preservation of children's mental health in a timely and adequate manner. Thus, parents need help and guidance too. In many districts of Serbia, new COVID-19 SOS telephone lines were opened to provide help to those in distress. Furthermore, many institutions and organizations, such as the WHO, CDC, and UNICEF, provide information and guidelines for parents. Providing space for children to express their fears freely and talk about their concerns also provides space for parents to create a safe and positive narrative through which they can communicate to their children that they are safe and understood.

### Limitations and Future Directions

One of the frequent limitations of research on the origin of children's fear is related to retrospective accounts. Our data were collected during the COVID-19 national state of emergency in Serbia, which enabled us to base our study on children's and parents' real-time reports about their fear related to COVID-19. Therefore, our data are arguably not prone to recall bias. Furthermore, a wide age range of children was covered. However, our study still suffers from various limitations. First of all, due to the small number of children who reported that they had been infected with COVID-19, we had to exclude this variable, which made it impossible to explore Rachman's model in its entirety. Future studies should focus on the effects of trauma exposure in children who have been infected with the virus and the psychological consequences that may ensue. It should be emphasized that some instruments are created by the authors for the purpose of this study. Although the internal consistency of these measures is satisfactory, due to the pandemic conditions (e.g., the importance of collecting data soon after the national state of emergency was declared), these instruments lack proper psychometric validation. An additional shortcoming lies in the fact that the data related to the gender of the parents were omitted. Thus, we do not know if the parents' sample consists predominantly of mothers, fathers, or both genders are equally represented. Due to the difference in the prevalence of mental health problems related to affective disorders [e.g., McLean et al. (2011)], and since it has been less frequently explored in previous research, the role of the fathers in the development of children's fear may be significant [see Bögels and Phares (2008), for more detail]. Also, the data related to the parents' gender could be significant for the interpretation of the results and instrumental in gaining additional insight into gender differences in negative information transmission or modeling. Finally, following the suggestion by Field and Storksen-Coulson (2007) that indirect pathways interact mutually, exploring the interactive effects of behavior and verbal negative information transmission may be highly relevant.

## Data Availability Statement

The raw data supporting the conclusions of this article will be made available by the authors, without undue reservation.

## Ethics Statement

The studies involving human participants were reviewed and approved by Institutional Review Board of the Department of Psychology, Faculty of Philosophy, University of Belgrade, Serbia. Written informed consent to participate in this study was provided by the participants' legal guardian/next of kin.

## Author Contributions

AR: research conception and design, instrument development, data collection, data analysis and interpretation, drafting the article (Sections Introduction, Results, and Discussion), and final approval of the version to be submitted. IM: research design, instrument development, data collection, data analysis and interpretation, drafting the article (Sections Method, Results, and Discussion), and final approval of the version to be submitted. SP: research design, instrument development, data collection, data interpretation, drafting the article (Sections Method, Results, and Discussion), and final approval of the version to be submitted. KK: research conception and design, data analysis and interpretation, drafting and revision of the manuscript, and final approval of the version to be submitted. All authors contributed to the article and approved the submitted version.

## Conflict of Interest

The authors declare that the research was conducted in the absence of any commercial or financial relationships that could be construed as a potential conflict of interest.
