# Supplementary material for: Don't Think That Kids Aren't Noticing: Indirect Pathways to Children's Fear of COVID-19
Source: Front Psychol. 2021 Mar 11;12:635952. doi: 10.3389/fpsyg.2021.635952 (PMC7991716; doi:10.3389/fpsyg.2021.635952)
Supplement: Supplementary file 1 [file Data_Sheet_1.pdf]

Supplementary Material

Supplementary Appendices

Supplementary Appendix A: Items of The Fear of COVID-19 Questionnaire for Children (FC19Q-C)

Items
I would be scared if I caught the Coronavirus.
I would be scared if someone I knew caught the Coronavirus.
I am scared that if I caught the Coronavirus, I would get sick really bad.
I am afraid that someone close to me might get really sick with the Coronavirus.
If I get sick with the Coronavirus, I will recover quickly.
It scares me that it might be very painful if I got sick with the Coronavirus.
I am afraid I might transmit the Coronavirus to someone.
It scares me that the Coronavirus is so tiny that it cannot be seen with the naked eye.
It happens that I dream about me or someone close to me getting the Coronavirus.
I keep thinking about whether or not I have the Coronavirus.
When I hear someone coughing, I immediately get scared that he or she has the Coronavirus.
I don't think I am going to be the one to get sick from the Coronavirus.
It's nothing so scary to get the Coronavirus.
Ever since this Coronavirus situation started, I'm more afraid of catching any disease in general.

Supplementary Appendix B: Items of NFITS, FITS and MODS

Non-Family Information Transmission Scale (NFITS)
While watching TV, I often come across news about how dangerous the Coronavirus is.
While chatting with my friends, I often find out disturbing information about the Coronavirus.
While talking with my teachers, they often say things about the Coronavirus that scare me.
Disturbing information about the Coronavirus often pop up on social networks (Facebook, Instagram, Twitter…). *
I saw posters and billboards about the Coronavirus on the street which caused me some discomfort.
Family Information Transmission Scale (FITS)
My parents warn me to beware of the Coronavirus even when I am at home and do not go outside.
My parents tell me how dangerous the Coronavirus is.
My parents draw my attention to bad things that could happen if one of us got sick with the Coronavirus.
Modeling Scale (MODS)
I notice from my parents' behavior that they are scared because of the Coronavirus.
My parents get upset when they watch TV news about the Coronavirus.
Every day, my parents talk to someone about the Coronavirus.

Since this Coronavirus situation started, my parents have been asking me more about my health.

My parents are trying to make good use of this time we are spending together while this Coronavirus situation lasts.

My parents sometimes make jokes about the Coronavirus.

I feel fear in my parents' voice when they talk about the Coronavirus.

My parents answer my questions about the Coronavirus calmly.

Ever since it became known that the Corona virus is present in Serbia as well, my parents have been more upset than before.

---

* It was suggested for all children to skip this question if they do not have any profile on social networks.
